# Evaluating the effect of drunk driving on fatal injuries among vulnerable road users in Taiwan: a population-based study

**DOI:** 10.1186/s12889-022-14402-3

**Published:** 2022-11-10

**Authors:** Hui-An Lin, Cheng-Wei Chan, Bayu Satria Wiratama, Ping-Ling Chen, Ming-Heng Wang, Chung-Jen Chao, Wafaa Saleh, Hung-Chang Huang, Chih-Wei Pai

**Affiliations:** 1 Department of Emergency Medicine, Taipei Medical University Hospital, Taipei City, 110 Taiwan; 2 Graduate Institute of Injury Prevention and Control, College of Public Health, Taipei Medical University, Taipei City, 110 Taiwan; 3 Department of Emergency Medicine, New Taipei City Hospital, New Taipei City, Taiwan; 4 College of Medicine, Chang Gung University, Taoyuan City, Taiwan; 5 Department of Emergency Medicine, Chang Gung Memorial Hospital, Taoyuan City, Taiwan; 6 Department of Epidemiology, Biostatistics, and Population Health, Faculty of Medicine, Public Health and Nursing, Universitas Gadjah Mada, Yogyakarta City, 55281 Indonesia; 7 Department of Traffic Management, Taiwan Police College, Taipei City, Taiwan; 8 Department of Traffic Science, Central Police University, Kueishan District, Taoyuan City, 33304 Taiwan; 9 Transport Research Institute, Edinburgh Napier University, Edinburgh, Scotland; 10 Division of General Surgery, Department of Surgery, Taiwan Adventist Hospital, Taipei City, Taiwan

**Keywords:** Drunk driving, Blood alcohol concentration, Fatal injury, Vulnerable road user

## Abstract

**Background:**

Most studies have focused on injuries sustained by intoxicated drivers themselves, but few have examined the effect of drunk driving on injury outcomes among VRUs (vulnerable road users) in developing countries. This study aims to evaluate the effect of drunk driving on fatal injuries among VRUs (pedestrians, cyclists, or motorcyclists).

**Methods:**

The data were extracted from the National Taiwan Traffic Crash Dataset from January 1, 2011, to December 31, 2019. Crashes involving one motorized vehicle and one VRU were considered. This study examines the effect of drunk driving by estimating multivariate logistic regression models of fatal injuries among VRUs after controlling for other variables.

**Results:**

Among 1,416,168 casualties, the fatality rate of VRUs involved in drunk driving was higher than that of general road users (2.1% vs. 0.6%). Drunk driving was a significant risk factor for fatal injuries among VRUs. Other risk factors for fatal injuries among VRUs included VRU age ≥ 65 years (adjusted odds ratio [AOR]: 5.24, 95% confidence interval [CI]: 5.53–6.07), a nighttime accident (AOR: 4.52, 95% CI: 4.22–4.84), and being hit by a heavy-duty vehicle (AOR: 2.83, 95% CI: 2.26–3.55). Subgroup analyses revealed a linear relationship between driver blood alcohol concentration (BAC) and the risk of fatal injury among motorcyclists. Motorcyclists exhibited the highest fatality rate when they had a BAC ≤ 0.03% (AOR: 3.54, 95% CI: 3.08–4.08).

**Conclusion:**

Drunk driving was associated with a higher risk of fatality for all VRUs. The risk of fatal injury among motorcyclists was linearly related to the BAC of the drunk drivers. Injuries were more severe for intoxicated motorcyclists, even those with BAC ≤ 0.03%, which is within the legal limit.

## Background

Alcohol acts as a central nervous system depressant that alters the level of consciousness [1, 2] and reduces the attentional and behavioral control of drivers [3, 4]. Drunk driving is a risky behavior; drunk drivers may judge the traffic condition improperly because they exhibit overestimation of personal abilities [5], excessive bravery [6], and a tendency to be affected by false memory [7]. Alcohol also interferes with visual acuity, perception, and psychomotor function; reduces reactions to impulses and environmental vigilance; and impairs the postural control of drivers [8–13]. Moreover, impaired decision-making [14] and information processing are evident among drivers with a positive blood alcohol concentration (BAC) [15]. Simulation studies have also demonstrated negative effects of alcohol on driving speed [16, 17], accelerating and braking behavior [18], and lane positioning [19].

The positive correlation between drunk driving and motor vehicle crashes (MVCs) has been well documented [20–34]. Even with a mildly elevated BAC (0.01–0.03%), drunk drivers cause more MVCs than do drivers who have not consumed alcohol [35–37]. Drunk driving not only increases the MVC risk but also results in more fatal crashes [28, 38, 39]. Zador et al. revealed that drivers with BACs < 0.1% contributed to more fatal injuries to both themselves and other road users [40]. Reynaud et al. analyzed the French police records through a 5-year period and found that 31.5% of those who died in an accident had a positive BAC, and 9.8% of them had a BAC over the legal limit [41]. The detrimental effect of alcohol use has also been confirmed in another French study [42], suggesting that fatigue, when combined with alcohol, presented a particularly high risk of crashes leading to death or serious injuries. Moreover, the victims of alcohol-impaired driving have higher risks of hospitalization, hypotension, Glasgow Coma Scale (GCS) scores, and events of cardiac arrest [28]. A retrospective analysis of 474 autopsy reports documenting fatalities in traffic crashes revealed 177 victims with a positive BAC [39]. Substance and alcohol use was also reported to be associated with reduced reaction times [43], as well as several risky behaviours such as driving without a seatbelt [44], unlicensed driving [45], and speeding [46].

In traffic accidents, vulnerable road users (VRUs)—motorcyclists, bicyclists, and pedestrians—sustain severe injuries and death at a higher rate than motorists. This is because without the protection afforded by a metal structure, VRUs generally sustain more severe injuries than car occupants [47]. Moreover, car drivers may have difficulty identifying or perceiving VRUs in traffic due to their being poorly visible and having a small size, which may increase the severity of a crash in the event of an accident [48–51].

Most studies have focused on injuries sustained by intoxicated drivers themselves, but few have examined the effect of drunk driving towards VRUs (vulnerable road users) in developing countries such as Taiwan. To fill this research gap, we analyzed Taiwan’s national police crash data and investigated the effects of drunk driving with other risk factors on fatal injuries among VRUs.

## Methods

### Study participants and data source

**Fig. 1:**
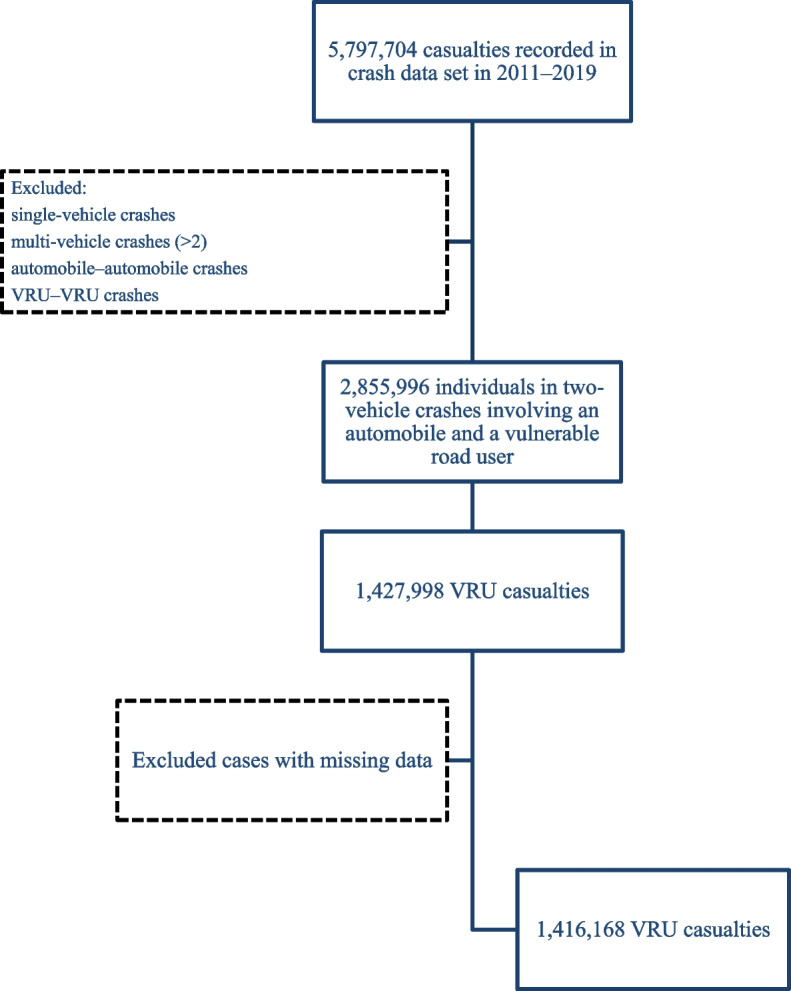
Selection of casualties

This study analyzed the National Taiwan Traffic Crash Dataset from January 1, 2011, to December 31, 2019. The dataset is administrated by the National Police Agency of Taiwan. Experienced police crash investigators are assigned to arrive at the scene and record the information which includes crash, vehicle, and victim files. Crash files contain data on road traffic crash characteristics such as time of crash, date of crash, weather condition, light condition, and various environmental factors (such as geographic location, speed limit, type and condition of road, and apparent distance). Vehicle files contain data on characteristics of the vehicle involved in the crash, such as first point of impact, type of vehicle, and vehicle maneuver. Furthermore, data on victim characteristics such as age, sex, injury severity level, license status, BAC, travel purpose, and restraint use are contained in the victim files. Similar to those in other countries, the Dataset is considered complete for multi-vehicle crashes but less complete for single-vehicle crashes; such an underreporting problem is less of a concern as the present study focuses on multi-vehicle crashes (i.e., an automobile and a VRU). In addition, variables that contain numerous missing data (e.g., hit-and-run crashes) or are unreliable (e.g., mobile phone use) were not considered in the current research. Every road traffic-related crash reported to the police was recorded in the dataset, which is maintained by the National Police Agency of Taiwan. In this study, we focused on crashes involving one automobile and one VRU (motorcyclist, cyclist, or pedestrian). Figure 1 illustrates the data extraction flowchart for this study. We excluded single-vehicle crashes, multiple-vehicle (> 2) crashes, VRU–VRU crashes, and crashes involving no VRUs from the dataset. Finally, we removed cases with missing data because we used a complete case analysis approach for our data analysis. This study was approved by the Joint Institutional Review Board of Taipei Medical University (number: N202007045). The current research analysed national crash data without individuals’ confidential information such as names or identity numbers. As a result, the Institutional Review Board affiliated with Taipei Medical University waived the informed consent.

### Study variables

Two injury severity levels were recorded: fatal injury (death within 24 h after crash) and nonfatal injuries (sustained injuries and survived for > 24 h). We also collected basic demographic data such as age, sex, participant’s safety behaviors, including helmet use by motorcyclists and bicyclists, BAC level, and the license status of drivers and motorcyclists. Because bicyclists and pedestrians are not required to be tested for alcohol use in the event of traffic accident, their BAC levels were not included in the present analysis.

Temporal variables included in this research were the time of the crash (rush hour, daytime, night, or early morning) and whether the accident occurred on a weekday or weekend. Rush hour was defined as 07:00 AM to 08:59 AM and 5:00 PM to 7:59 PM, daytime was defined as 09:00 AM to 4:59 PM, evening was defined as 8:00 PM to 11:59 PM, and nighttime was defined as 12:00 AM to 06:59 AM.

The following road and environmental factors were analyzed: weather (fine weather refers to sunny and cloudy days; adverse weather includes rainy, snowy, foggy, or sandy conditions and strong winds), and light conditions (no light at night, illuminated at night, morning or dawn, and daytime with natural light; if the incident was in a tunnel or underpass, the setting was deemed night). Taiwan has six municipalities: Kaohsiung, New Taipei, Taichung, Tainan, Taipei, and Taoyuan. Other regions are defined as counties. Several road conditions were considered in this study, including road type (crossroad or not), road surface conditions (slippery road includes snowy/icy, oily, muddy, or damp road), road defect (intact road surface or a defective road, meaning soft terrain, uneven road, or road with pit or hole), and driver’s sightline (clear sight or obstacle in sight). Speed limit was divided into less than 50 km/h and ≥ 50 km/h. Injured body regions of VRUs were categorized as a head and neck injury and other injuries including the chest, abdomen, back, pelvis, and extremities. Table 1 illustrates variables included in analysis.

**Table 1:** Description of each variable

Variable	Description
Sex of drivers	male, female
Sex of VRUs	as above
Age of drivers	0–17, 18–40, 41–64, and ≥ 65 y
Age of VRUs	as above
Day of week	• weekdays: Monday to Friday
	• weekend: Saturday and Sunday
Time period	• rush hour: 07:00 AM to 08:59 AM; 17:00 PM to 19:59 PM
	• daytime: 09:00 AM to 16:59 PM
	• evening: 20:00 PM to 23:59 PM
	• night: 00:00 AM to 06:59 AM
Municipality	• municipality: Taipei, New Taipei, Taoyuan, Taichung, Tainan, Kaohsiung
	• county: cities other than the six municipality
Speed limit	2 categories: < 50 km/h and ≥ 50 km/h
Weather	• fine: sunny
	• adverse: cloudy, rainy, snowy, foggy, strongly windy, sand blown by the wind, or stormy
Light condition	• natural day light
	• dawn or twilight
	• night (or in tunnel/underpass) with illumination
	• night (or in tunnel/underpass) without illumination
Road type	• not crossroad (single-way, circle, and square)
	• crossroad (T/Y-intersection, crossroad, and multiple-way)
Road surface	• dry surface
	• slippery surface: frosty, oily, muddy, or wet
Road defect	• intact: no defect of the road surface
	• defect: soft terrain, protuberance, potholes
Sight obstacle	• clear sight: no obstacle in sight
	• sight obstacle: curve, ramp, building, trees or crops, vehicles, and other materials could affect driver’s sight
BAC of drivers	nil, 0.01 ≤ BAC ≤ 0.03, 0.031% ≤ BAC ≤ 0.05, 0.051% ≤ BAC ≤ 0.08, 0.081% ≤ BAC ≤ 0.11%, BAC > 0.11%
BAC of VRUs	as above
License status of drivers	• licensed: qualified license
	• unlicensed: no license, revoked or inappropriate license
License status of motorcyclists	As above
Automobile type	• heavy-duty vehicle: truck, bus, trailer, tractor
	• passenger car
	• special car: military vehicle, ambulance, fire engine, police vehicle, tracked engineer
VRU type	motorcyclist, bicyclist, and pedestrian
Injured body region of VRUs	• head and neck
	• other parts
Protective device of VRUs	Helmeted and unhelmeted

### Statistical analysis

We first compared the distribution of fatal injuries by demographic factors, behaviors, vehicle attributes, crash characteristics, environmental factors, time factors, and crash types. A *p* value < 0.2 was used as the cutoff point to incorporate risk factors into multivariate analysis. Multiple logistic regression analysis with backward selection was used to calculate the adjusted odds ratios (AORs). Multicollinearity was assessed using Cramer’s V and the chi-square independent test. A subgroup analysis was conducted separately for motorcyclists, bicyclists, and pedestrians. A full model (automobile VRUs) was first estimated, followed by three additional models: an automobile–motorcycle (A-M) model, an automobile–bicycle (A-B) model, and an automobile–pedestrian (A-P) model. Statistical significance was defined as *p* < 0.05. The binary logistic regression model has been broadly utilized in the field of medicine and trauma [52–54] to identify the significant risk factors of the dichotomous outcome. In binary logistic regression model, the dependent variable is not limited by the assumptions of a continuous or normal distribution.

In the binary logistic regression model, the equation is formulated as follows:

$$g(x)=\beta 0+{\beta}_1{x}_1+{\beta}_2{x}_2+\dots +{\beta}_p{x}_p$$

where x_j_ is the value of the jth independent variable, β_j_ is the corresponding coefficient for j = 1, 2, 3,. .., p, and p is the number of independent variables.

The conditional probability of a positive outcome given the independent variable is as follows:

$$\pi (x)=\frac{\exp \left(g(x)\right)}{1+\exp \left(g(x)\right)}$$

The maximum likelihood method was used to estimate the parameters of the logistic regression model by constructing the likelihood function:

$$I\left(\beta\right)=\prod\limits_{i=1}^n\pi\left(x_i\right)^{y_i}\left(1-\mathrm\pi\left({\mathrm x}_{\mathrm i}\right)\right)^{1-y_i}$$

where y_i_ denotes the ith observed outcome with a value of either 0 or 1 and i = 1, 2, 3,. .., n, where n is the number of observations. The best regression estimation of β was determined by maximizing the log-likelihood expression:

$$LL\left(\beta \right)=\ln \left(l\left(\beta \right)\right)=\sum\limits_{i=1}^n\left\{{y}_i\ln \left(\uppi \left({x}_i\right)\right)+\left(1-{y}_i\right)\ln \left(1-\uppi \left({x}_i\right)\right)\right\}$$

The exponentiated coefficient exp(*β*_*j*_), odds ratio (OR), demonstrates the effect of attributes on the likelihood of fatal injuries in logistic regression model, with a 95% confidence interval (CI) of (exp(*β*_*j*_ − 1.96*sβ*_*j*_), exp(*β*_*j*_ + 1.96*sβ*_*j*_)), where *sβ* is the standard error of coefficient *β*. An OR of > 1 indicated a positive association between the target independent variable and fatal injuries, whereas an OR of < 1 indicated a negative association between the interest attribute and fatal injuries. An OR of 1 indicated that no association was found between the interest attributes and outcomes. If there were missing data, we conducted a sensitivity analysis to compare data with and without missing data by using the chi-square test. We used IBM SPSS Statistics for Windows, Version 22.0. Armonk, NY: IBM Corp to perform the statistical analysis.

## Results

A total of 5,797,704 victims involved in traffic accidents were documented by police from 2011 to 2019. After applying the exclusion criteria, 2,855,996 casualties remained in the automobile–VRU crash category. Half of the casualties in automobile–VRU crashes were VRUs, and the other half were automobile drivers; accordingly, 1,427,998 VRU casualties were included in our analysis. After excluding missing data, 1,416,168 casualties with intact records were analyzed. Figure 1 illustrates the data extraction flowchart for this study. A total of 5,797,704 victims involved in traffic accidents were documented by police from 2011 to 2019. We excluded single-vehicle crashes, multiple-vehicle (> 2) crashes, VRU–VRU crashes, and crashes involving no VRUs from the dataset. Furthermore, we removed cases with missing data because we used a complete case analysis approach for our data analysis. Finally, 1,416,168 casualties with intact records were analyzed.

Table 2 presents the distribution of injury severity across a set of independent variables. Both being hit by a male driver and being a male VRU were associated with higher rates of fatal injuries to VRUs (both were 0.7%). VRUs aged ≥65 years had a higher mortality rate than other age groups. Higher than at other times, 2.9% of fatal injuries occurred at night. Regions outside a municipality (0.8%) and with a speed limit over 50 km/h (0.6%) were associated with a higher rate of fatal injuries. Nighttime with unlit streets was associated with a higher mortality rate (2.4%) than daytime. Fatal injuries were less prevalent under some road conditions, such as being a crossroad (0.6% vs. not crossroad 0.7%), a slippery road surface (0.5% vs. dry surface 0.6%), and an unobstructed view of the road (0.6% vs. with sight obstacle 1.0%). Weather did not significantly affect fatality rates. A positive BAC and a driver being unlicensed were associated with higher rates of fatal injuries among VRUs. Furthermore, the rates of fatal injuries were higher in crashes with buses and trucks (4.1%) and when the casualties were pedestrians (2.7%); bicyclists (1.6%) and motorcyclists (0.5%) had lower fatality rates. Notably, motorcyclists accounted for 91.8% of all VRUs involved in vehicle–VRU crashes. VRUs who sustained head and neck injuries had higher mortality rates (7.2%) compared with VRUs with other injured regions (0.4%).

**Table 2:** Distribution of injury severity among VRUs by a set of independent variables

	Total	Nonfatal (*n* = 1,267,684)	Fatal injuries (*n* = 7774)	*P* value
**Demographic factors**				
Sex of drivers				
Male	958,978	99.3%	0.7%	< 0.001
Female	316,480	99.7%	0.3%	
Sex of VRUs				
Male	722,095	99.3%	0.7%	< 0.001
Female	553,363	99.5%	0.5%	
Age of drivers				
0–17	829	98.8%	1.2%	0.143
18–40	568,875	99.4%	0.6%	
41–64	621,631	99.4%	0.6%	
≥ 65	77,535	99.4%	0.6%	
Age of VRUs				
0–17	41,256	99.5%	0.5%	< 0.001
18–40	748,472	99.7%	0.3%	
41–64	333,323	99.3%	0.7%	
≥ 65	150,147	97.9%	2.1%	
**Temporal factors**				
Day of week				
Weekdays	966,334	99.4%	0.6%	0.355
Weekend	309,124	99.4%	0.6%	
Time period				
Rush hour	442,308	99.5%	0.5%	< 0.001
Daytime	610,555	99.5%	0.5%	
Evening	153,987	99.5%	0.5%	
Night	68,608	97.1%	2.9%	
**Environmental factors**				
Municipality				
County	558,931	99.2%	0.8%	< 0.001
Municipality	716,527	99.5%	0.5%	
Speed limit				
< 50 km/h	293,687	99.5%	0.5%	< 0.001
≥ 50 km/h	981,771	99.4%	0.6%	
Weather				
Fine	1,013,085	99.4%	0.6%	0.479
Adverse	262,313	99.4%	0.6%	
Light condition				
Natural day light	913,194	99.5%	0.5%	< 0.001
Dawn or twilight	39,679	99.0%	1.0%	
Night with illumination	311,240	99.3%	0.7%	
Night without illumination	11,339	97.6%	2.4%	
Road type				
not crossroad	430,745	99.3%	0.7%	< 0.001
Crossroad	844,713	99.4%	0.6%	
Road surface				
Dry surface	1,105,198	99.4%	0.6%	< 0.001
Slippery surface	170,260	99.5%	0.5%	
Road defect				
Intact	1,271,147	99.4%	0.6%	0.036
Defect	4311	99.1%	0.9%	
Sight obstacle				
Clear sight	1,239,138	99.4%	0.6%	< 0.001
Sight obstacle	36,320	99.0%	1.0%	
**Behavior factors**				
BAC of drivers				
nil	1,251,862	99.4%	0.6%	< 0.001
.01–0.03%	7663	98.2%	1.8%	
0.031–0.05%	2970	98.2%	1.8%	
0.051–0.08%	3179	97.8%	2.2%	
0.081–0.11%	2939	97.8%	2.2%	
> 0.11%	6854	97.5%	2.5%	
License status of drivers				
Licensed	1,248,719	99.4%	0.6%	< 0.001
Unlicensed	26,739	98.4%	1.6%	
**Crash factors**				
Automobile type				
Heavy-duty vehicle	44,673	95.9%	4.1%	< 0.001
Passenger car	1,223,860	99.5%	0.5%	
Special car	6925	98.7%	1.3%	
VRU type				
Motorcyclist	1,170,600	99.5%	0.5%	< 0.001
Bicyclist	50,149	98.4%	1.6%	
Pedestrian	54,709	97.3%	2.7%	
Injured body region of VRUs				
Other parts	1,229,596	99.6%	0.4%	< 0.001
Head and neck	45,862	92.8%	7.2%	

Table 3 presents the results of the multivariate logistic models of fatal injuries. Male drivers (AOR: 1.55, 95% confidence interval [CI]: 1.45–1.67) and male VRUs (AOR: 1.63, 95% CI: 1.55–1.72) were both associated with higher risks of fatalities. VRUs aged ≥65 years were over 5 times more likely to sustain fatal injuries (AOR: 5.24, 95% CI: 4.53–6.07) than were younger groups. Fatal injuries among VRUs were more prevalent at nighttime (AOR: 4.52, 95% CI: 4.22–4.84) and in dark environments without illumination (AOR: 2.37, 95% CI: 2.05–2.75). When the travel speed was considered, counties rather than municipalities (AOR: 1.22, 95% CI: 1.16–1.28) and a speed limit ≥50 km/h (AOR: 1.29, 95% CI: 1.21–1.37) both contributed to higher likelihoods of fatal injuries. When the road surface was dry (AOR: 1.34, 95% CI: 1.24–1.44) and driver sight was obstructed (AOR: 1.39, 95% CI: 1.24–1.56), VRUs also had an additional risk of fatal injuries. Road type and road defects were not significant risk factors in multivariate analysis. Alcohol use among drivers was associated with an increased likelihood of fatal injuries to VRUs compared with alcohol nonuse. Drivers with an alcohol level ≥ 0.08% were associated with a higher VRU fatality risk (BAC 0.08–0.11%, AOR: 2.79, 95% CI: 2.14–3.63; BAC ≥ 0.11%, AOR: 2.73, 95% CI: 2.30–3.23). Unlicensed driving (AOR: 2.03, 95% CI: 1.82–2.26) and the accident involving a truck or bus (AOR: 2.82, 95% CI: 2.26–3.55) also appeared to be independent risk factors for deaths. Pedestrians (AOR: 2.17, 95% CI: 2.02–2.32) had a higher mortality rate than did other VRUs. VRUs with head and neck injuries were 12 times more likely to have fatal injuries (AOR: 12.38, 95% CI: 11.78–13.02).

**Table 3:** Multivariate logistic regression of VRU fatalities and potential risk factors

	β	AOR (95% CI)	*P* value
**Sex of drivers**			
Male (vs. female)	0.44	1.55 (1.45–1.67)	< 0.001
**Sex of VRUs**			
Male vs. female	0.49	1.63 (1.55–1.72)	< 0.001
**Age of VRUs**			
0–17	–	1.00 (reference)	–
18–40	0.04	1.04 (0.90–1.21)	0.598
41–64	0.82	2.26 (1.95–2.63)	< 0.001
≥ 65	1.66	5.24 (4.53–6.07)	< 0.001
**Time period**			
Rush hour	–	1.00 (reference)	–
Daytime	0.01	0.99 (0.93–1.06)	0.802
Evening	0.23	1.25 (1.14–1.37)	< 0.001
Night	1.51	4.52 (4.22–4.84)	< 0.001
**Municipality**			
County (vs. municipality)	0.20	1.22 (1.16–1.28)	< 0.001
**Speed limit**			
≥ 50 km/h	0.25	1.29 (1.21–1.37)	< 0.001
**Light**			
Natural day light	–	1.00 (reference)	–
Dawn or twilight	0.02	0.98 (0.87–1.11)	0.780
Night with illumination	0.18	1.20 (1.12–1.29)	< 0.001
Night without illumination	0.86	2.37 (2.05–2.75)	< 0.001
**Road type**			
Not crossroad (vs. crossroad)	0.05	1.05 (1.00–1.10)	0.072
**Road surface**			
Dry road (vs. conditional)	0.29	1.34 (1.24–1.44)	< 0.001
**Road defect**			
Defect (vs. intact)	0.32	1.37 (0.97–1.93)	0.072
**Sight obstructed**			
Sight obstructed (vs. good sight)	0.33	1.39 (1.24–1.56)	< 0.001
**BAC of drivers**			
nil	–	1.00 (reference)	–
0.01–0.03%	0.76	2.14 (1.79–2.58)	< 0.001
0.031–0.05%	0.68	1.97 (1.46–2.65)	< 0.001
0.05–0.08%	0.76	2.06 (1.58–2.69)	< 0.001
0.08–0.11%	1.03	2.79 (2.14–3.63)	< 0.001
> 0.11%	1.00	2.73 (2.30–3.23)	< 0.001
**License status of drivers**			
Unlicensed	0.71	2.03 (1.82–2.26)	< 0.001
**Automobile type**			
Heavy-duty vehicle	1.04	2.83 (2.26–3.55)	< 0.001
Passenger car	−0.71	0.49 (0.39–0.61)	< 0.001
Special car	–	1.00 (reference)	–
**VRU type**			
Motorcyclist	–	1.00 (reference)	–
Bicyclist	0.29	1.33 (1.23–1.45)	< 0.001
Pedestrian	0.77	2.17 (2.02–2.32)	< 0.001
**Injured body region of VRUs**			
Head and neck	2.52	12.38 (11.78–13.02)	< 0.001
AUC (95%CI)	0.913 (0.910–0.917)		

Table 4 presents the results of the subgroup analysis by VRU category. Drivers with a positive BAC were associated with higher odds of fatal injuries in all VRU groups. Driver BAC had a linear relationship with fatality risk among motorcyclists but not among bicyclists or pedestrians. Motorcyclists had the highest risk of death when their alcohol level was as low as 0.01–0.03% (AOR: 3.54, 95% CI: 3.08–4.08). Unlicensed riders also had a higher risk of fatalities (AOR: 1.71, 95% CI: 1.59–1.84). With regard to the effect of unlit darkness, the magnitude of the increased risk of fatal injury was the highest for pedestrians (AOR: 3.57, 95% CI: 2.68–4.76), followed by that for bicyclists (AOR: 2.66, 95% CI: 1.77–3.99) and motorcyclists (AOR: 1.55, 95% CI: 1.27–1.89).

**Table 4:** Subgroup analysis: fatalities of different VRUs

	Motorcyclists		Bicyclists		Pedestrians	
	AOR (95%CI)	*P* value	AOR (95%CI)	*P* value	AOR (95%CI)	*P* value
**Age of VRUs**						
0–17	1.00 (reference)	–	1.00 (reference)	–	1.00 (reference)	–
18–40	1.08 (0.90–1.31)	0.420	2.26 (1.45–3.53)	< 0.001	1.10 (0.73–1.65)	0.653
41–64	2.06 (1.70–2.49)	< 0.001	5.10 (3.48–7.49)	< 0.001	2.87 (2.00–4.11)	< 0.001
≥ 65	4.26 (3.53–5.15)	< 0.001	11.06 (7.66–15.96)	< 0.001	7.51 (5.31–10.64)	< 0.001
**Light**						
Natural daylight	1.00 (reference)	–	1.00 (reference)	–	1.00 (reference)	–
Dawn or twilight	0.80 (0.68–0.93)	0.004	1.47 (1.08–1.99)	0.014	1.22 (0.95–1.57)	0.116
Night with illumination	0.89 (0.82–0.97)	0.011	1.44 (1.15–1.80)	0.002	1.85 (1.58–2.18)	< 0.001
Night without illumination	1.55 (1.27–1.89)	< 0.001	2.66 (1.77–3.99)	< 0.001	3.57 (2.68–4.76)	< 0.001
**Protective device of VRUs**						
No helmet	1.95 (1.83–2.09)	< 0.001	2.50 (1.86–3.35)	< 0.001	–	–
**BAC of drivers**						
nil	1.00 (reference)	–	1.00 (reference)	–	1.00 (reference)	–
0.01–0.03%	1.96 (1.57–2.44)	< 0.001	2.15 (1.19–3.89)	0.012	2.19 (1.42–3.36)	< 0.001
0.031–0.05%	1.68 (1.16–2.45)	0.006	2.21 (0.98–5.00)	0.057	2.10 (1.05–4.19)	0.035
0.051–0.08%	2.06 (1.49–2.87)	< 0.001	1.88 (0.87–4.05)	0.107	2.18 (1.26–3.78)	0.006
0.081–0.11%	2.90 (2.11–3.98)	< 0.001	3.74 (1.87–7.47)	< 0.001	1.55 (0.75–3.23)	0.237
> 0.11%	3.02 (2.45–3.73)	< 0.001	1.70 (0.97–2.96)	0.064	2.63 (1.87–3.68)	< 0.001
**BAC of VRUs**						
nil	1.00 (reference)	< 0.001	–	–	–	–
0.01–0.03%	3.54 (3.08–4.08)	< 0.001	–	–	–	–
0.031–0.05%	1.63 (1.18–2.26)	0.003	–	–	–	–
0.051–0.08%	1.68 (1.27–2.23)	< 0.001	–	–	–	–
0.081–0.11%	1.31 (0.98–1.74)	0.072	–	–	–	–
> 0.11%	1.62 (1.44–1.82)	< 0.001	–	–	–	–
**License status of drivers**						
Licensed	1.00 (reference)	–	1.00 (reference)	–	1.00 (reference)	–
Unlicensed	2.05 (1.79–2.34)	< 0.001	2.29 (1.65–3.17)	< 0.001	1.59 (1.23–2.04)	< 0.001
**License status of VRUs**						
Licensed	1.00 (reference)	–	–	–	–	–
Unlicensed	1.71 (1.59–1.84)	< 0.001	–	–	–	–
**Injured body region of VRUs**						
Other parts	1.00 (reference)	–	1.00 (reference)	–	1.00 (reference)	–
Head and neck	14.11 (13.30–14.97)	< 0.001	7.50 (6.42–8.76)	< 0.001	5.91 (5.25–6.64)	< 0.001
AUC (95% CI)	0.907 (0.903–0.912)		0.910 (0.898–0.921)		0.856 (0.846–0.867)	

## Discussion

Our study demonstrated that VRUs had additional risks of fatal injuries caused by drunk drivers after controlling for other variables. The higher the alcohol concentration of the driver was, the worse the fatality rates for the VRUs were, and this conclusion is in line with previous research [55–58]. A linear relationship was noted between driver BAC and the risk of fatalities among motorcyclists but not among cyclists and pedestrians. Such effects are likely attributed to several dimensions. First, in spite of speed data were not available in the Dataset, motorcyclists are generally moving much faster than those cycling or walking, thereby in turn leading to more devastating crash impacts [59–62], less reaction time [63], and high tendencies to lose control [64]. High traveling speed of motorcycles, relative to other VRUs, may act synergistically with driver BAC to increase injury severity. Such a linear relationship is likely due to the traffic exposure: fewer cyclists and pedestrians, compared with motorcycles, travel on roadways with higher speed limits. Our conjecture here needs to be ascertained in future research with additional data on crash locations and speed. While drunk driving appeared to be the main risk factors for fatal injuries among vulnerable road users, other studies [65] pointed out that mobile phone use may compromise pedestrians’ safety. Due to a lack of reliable data on mobile phone use, we identify this as a fruitful area for future studies.

Our data also highlighted that drunk riding increases motorcyclists’ mortality rate, concordant with previous research [66]. Notably, motorcyclists experienced the highest fatality rate at a legal BAC level (0.01–0.03%). In contrast to drivers, riders had the peak of fatality rate in a relative low BAC, and one early study also concluded that a low BAC level was associated with more crashes in motorcyclists than in drivers [67]. The relation between the risk of motorcyclists and their low BAC could attribute to the complexity of motorcycling, which requires concentration, balance, control and precision of movement through curves, and familiarity with the operation of the motorcycle [68]; these skills, especially balance, can be impaired at even a low alcohol concentration [69, 70]. Creaser and colleagues suggested that although riders with a low BAC preserved their cognitive and visual ability, they had to concentrate more on maintaining their riding balance, thereby sacrificing attention to cornering and hazard perception [68].

Traveling at night is generally considered risky due to poor visibility [56, 71–76]. In our data, VRUs had the highest risk of fatalities during night hours (00:00 AM to 06:59 AM), and pedestrians exhibited an additional increment in fatalities in this time frame. Compared with motorcycles and bicycles, pedestrians usually have less or no lightning instruments or reflectors, and drivers are prone to miss them in dim light. Furthermore, pedestrians also are smaller in size than other road users (i.e., machines), making them more difficult to be observed at night [77]. Appropriate measures to prevent crashes in dark environments include enhancing VRUs’ visibility through the use of lighting equipment or reflective clothes.

Head and neck injuries are common in fatal traffic crashes [78–84] and were associated with higher risks of death among all VRUs in our study. Helmet use reduced the fatality rate and demonstrated significant protective effects both among motorcyclists and bicyclists. The head is the only region of a rider that can be protected by a device, such as a helmet, and the benefit of a helmet in reducing injury severity and fatalities has been well documented [74, 78, 79, 81, 83]. Moreover, riding without a helmet has also been associated with other risky behaviors, such as drunk riding; both risky behaviors may lead to fatalities [85, 86]. Motorcyclists have an elevated risk of fatalities when drunk riding without helmets [52]. Although the number of head injuries in Taiwan has significantly decreased after helmet use by motorcyclists was mandated in 1997 [87], no legislation mandates the same for cyclists to date. Promoting helmet use among cyclists is clearly a public health issue.

To our knowledge, few studies have focused on the association between alcohol-impaired driving and VRU fatality. We analyzed the effect of alcohol-impaired driving on fatal injuries of VRUs and the individual risk of motorcyclists, bicyclists, and pedestrians. Our research represents to a contribution to profession through the insight that drunk driving among car drivers resulted in additional risks of mortality among all VRUs. Furthermore, a linear relationship was found between driver BAC and motorcyclist fatality rate. Interestingly, intoxicated motorcyclists, even with a BAC within the legal limit of 0.03%, had the highest rate of fatal injuries. Accordingly, we recommend several measures to improve the road safety. First, campaign for alcohol zero tolerance should be promoted to all population. Second, helmet use should not only be mandatory to motorcyclists, but also be promoted to bicyclists to reduce fatal head injuries. Furthermore, the high proportion of fatalities at nights, especially in unlit conditions, underscores the importance to enhance illumination instrument in areas where there are motorcyclists, bicyclists, and pedestrians. Last, VRUs, especially elderly bicyclists and pedestrians, may consider enhancing their own conspicuity at nights by using reflectors. However this paper is not without its limitations. First, vehicle speed was not available from the police crash records. Vehicle speed, instead of the surrogate variable “speed limit” used in the current research, may provide additional insights into fatalities. Second, detailed information on geometric factors, such as curvature or road alignments, that may play a crucial role in fatalities was not readily available from the police crash reports. Third, data on casualties who died at crash scenes were not available, and as a result, their BAC was not measured. These limitations may have underestimated the effect of drunk driving on fatalities among VRUs.

## Conclusion

Drunk driving results in additional risks of mortality among all VRUs, and a linear relationship was found between driver BAC and motorcyclist fatality rate. Intoxicated motorcyclists, even with a BAC within the legal limit of 0.03%, had the highest rate of fatal injuries. The results obtained in this current research endorse a tightened legislation for alcohol concentration limit in order to prevent fatal injuries among the vulnerable road users.

## Data Availability

The police-reported crash data, which are open to the researchers in Taiwan, are available from the Health and Welfare Data Science Center (http://dep.mohw.gov.tw/DOS/np-2497-113.html). Only citizens of Taiwan who fulfill the requirements of conducting research projects are eligible to apply for the police-reported crash dataset. The use of police-reported crash dataset is limited to research purposes only. Applicants must follow the Computer Processed Personal Data Protection Law.

## Abbreviations

BAC: Blood-alcohol concentration
MVCs: Motor vehicle crashes
GCS: Glasgow Coma Scale
VRU: Vulnerable road user
AORs: Adjusted odds ratios
CI: Confidence interval
